# Adiponectin Deficiency Enhances Anti-Tumor Immunity of CD8^+^ T Cells in Rhabdomyosarcoma Through Inhibiting STAT3 Activation

**DOI:** 10.3389/fonc.2022.847088

**Published:** 2022-04-22

**Authors:** Jiao Peng, Haifeng Huang, Qiuchan Huan, Chenghui Liao, Zebin Guo, Die Hu, Xiangchun Shen, Haitao Xiao

**Affiliations:** ^1^ Department of Pharmacy, Peking University Shenzhen Hospital, Shenzhen, China; ^2^ The High Efficacy Application of Natural Medicinal Resources Engineering Center of Guizhou Province, School of Pharmaceutical Sciences, Guizhou Medical University, Guiyang, China; ^3^ School of Pharmaceutical Sciences, Health Science Center, Shenzhen University, Shenzhen, China; ^4^ School of Pharmaceutical Science, Chongqing Medical University, Chongqing, China; ^5^ School of Pharmaceutical Science, Harbin Medical University, Heilongjiang, China

**Keywords:** CD8^+^ T cells, adiponectin, rhabdomyosarcoma, anti-tumor immunity, STAT3

## Abstract

Restoring the tumor-killing function of CD8^+^ T cells in the tumor microenvironment is an important strategy for cancer immunotherapy. Our previous study indicated that adiponectin (APN) deficiency reprogramed tumor-associated macrophages into an M1-like phenotype to inhibit rhabdomyosarcoma growth. However, whether APN can directly regulate the anti-tumor activity of CD8^+^ T cells remains unknown. In the present study, our results showed that exogenous APN inhibited *in vitro* CD8^+^ T cell migration as well as cytokines IFN-γ and TNF-α production. APN deficiency *in vivo* strengthened CD8^+^ T cell activation and cytotoxicity to restrain rhabdomyosarcoma, evidenced by an increase in the expression of IFN-γ and perforin in CD8^+^ T cells and the frequency of CD8^+^IFN-γ^+^ T cells in the spleen and lymph nodes, as well as increasing cytokine production of IFN-γ, perforin, TNF-α, and decreasing cytokine production of IL-10 in the serum. Mechanistically, STAT3 was identified as a target of APN in negatively regulating the anti-tumor activity of CD8^+^ T cells. *In vivo*, a STAT3 inhibitor remarkably increased CD8^+^ as well as CD8^+^IFN-γ^+^ T cells in the spleen and lymph nodes. Taken together, we substantiated that APN deficiency directly maintains the activation of CD8^+^ T cells to inhibit rhabdomyosarcoma growth by suppressing STAT3 activation, indicating a promising APN-based therapy for the treatment of rhabdomyosarcoma.

## Introduction

Rhabdomyosarcoma (RMS) is a high-grade malignant soft tissue sarcoma that includes embryonal rhabdomyosarcoma (ERMS) and alveolar rhabdomyosarcoma (ARMS) (1). It commonly occurs in children and teenagers, with an estimated incidence rate of 4.4 cases per 1 million in individuals under 20 years of age, accounting for 3% of pediatric cancers diagnosed annually (1, 2). Current clinical treatment of RMS is based on multimodal therapies, including surgery and chemotherapy, with or without radiotherapy (3). During the last few decades, progressive advancements in RMS treatment have made the overall survival rate up to 60%, but the overall survival rate of metastatic and relapsed/refractory RMS is only 10% to 20% (4, 5). Therefore, there is a clear need to develop therapeutic strategies for RMS. Recent mechanistic advances in immunology have identified that the immunosuppressive tumor microenvironment is the basis for tumor growth and its malignant properties since the host fails to induce an effective immune response (6). Hence, a therapeutic strategy that can counteract the immunosuppressive tumor microenvironment may be an alternative option for RMS.

Adiponectin (APN), a well-known adipocyte-secreted cytokine, has been shown to possess multiple immunoregulatory effects in both innate and adaptive immune systems. APN is capable of inducing anti-inflammatory cytokines, such as IL-10, IL-1RA, and IL-4, and suppressing pro-inflammatory cytokines, such as IL-1β, IL-6, and TNF-α from monocytes, macrophages, and dendritic cells (7–14). APN suppresses the cellular maturation of dendritic cells (7, 15), inhibits pro-inflammatory cytokines (IL-1β, IL-2, IL-8, TNF-α, IFN-γ) from T cells (7, 16), and induces T cell apoptosis and expansion of Tregs (15, 16). In our previous study, we found that APN was highly expressed in infantile RMS, especially in ARMS, one malignant subtype of RMS (17). APN deficiency could inhibit the growth of rhabdomyosarcoma in mice by reprogramming tumor-associated macrophages into an M1-like phenotype *via* suppressing p38 MAPK phosphorylation (17). We also found an extensive infiltration of CD8^+^ T cells in tumor tissues (17). However, whether APN can directly regulate the anti-tumor effect of CD8^+^ T cells remains unknown. In this study, we investigated the role APN on CD8^+^ T cells in the tumor microenvironment of rhabdomyosarcoma-bearing mice, and found that APN deficiency inhibited rhabdomyosarcoma growth, which was associated with enhancing the anti-tumor response of CD8^+^ T cells that infiltrated rhabdomyosarcoma through the inhibition of STAT3 activation.

## Results

### Exogenous APN Inhibits CD8^+^ T Cell Activation and Migration

**Figure 1:**
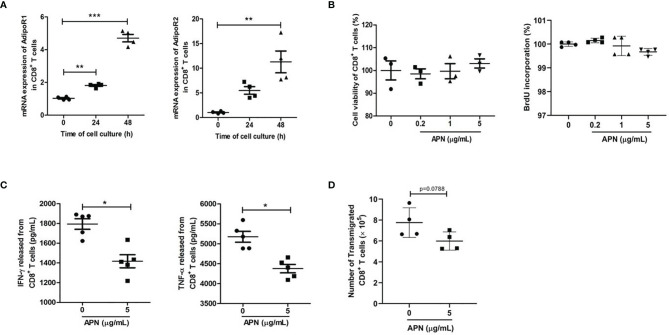
Exogenous APN inhibits CD8^+^ T cell activation and migration. **(A)** mRNA expression of AdipoR1 and AdipoR2 in inactive and activated CD8^+^ T cells (n = 4). **(B)** The effect of APN on CD8^+^ T cell proliferation (n =3-4). **(C)** The effect of APN on IFN-γ and TNF-α production from CD8^+^ T cells (n =5). **(D)** The effect of APN on CD8^+^ T cell migration (n=4). Data are expressed as the mean ± SEM from one of two or three separate experiments with same results. *p<.05, **p<.01 and ***p<.001.

To study the effect of APN on CD8^+^ T cells, we first detected the expressions of APN receptors, AdipoR1 and AdipoR2, in inactive and activated CD8^+^ T cells. The results revealed that these two receptors were expressed in CD8^+^ T cells, and their expression was upregulated after TCR activation (**Figure 1A**), which was consistent with a previous report (16). Subsequently, we stimulated purified CD8^+^ T cells with a combination of plate-bound anti-CD3 mAb and anti-CD28 mAb in the presence or absence of APN. The production of IFN-γ and TNF-α from CD8^+^ T cells significantly declined after APN treatment (5 μg/mL), but no obvious proliferative effect was observed (**Figures 1B, C**). The effect of APN on the migration of CD8^+^ T cells was also investigated using a transwell migration assay, which showed that the number of transmigrated CD8^+^ T cells obviously decreased after APN treatment (5 μg/mL) (**Figure 1D**). These data suggest that exogenous APN can inhibit CD8^+^ T cell activation and migration.

### APN Deficiency Inhibits Rhabdomyosarcoma Growth and Negatively Regulates the Anti-Tumor Effect of CD8^+^ T Cells in Tumors

**Figure 2:**
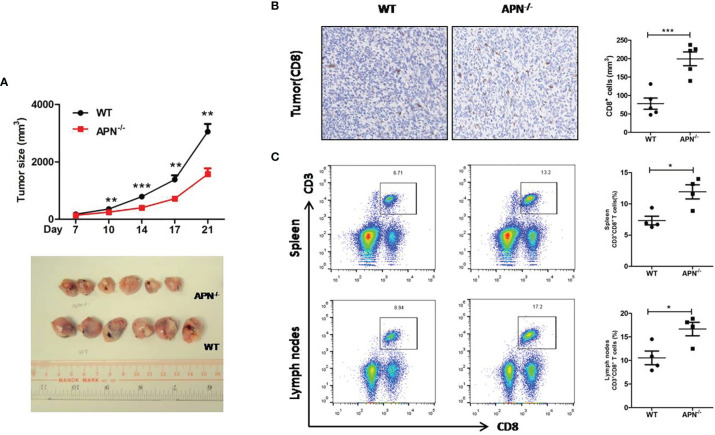
APN deficiency inhibits rhabdomyosarcom growth and increases the accumulation of CD8^+^ T cells in tumor mass, spleen and lymph nodes. **(A)** Tumor size of wild type (WT) and in APN deficiency (APN^−/−^) mice (n=6 for each group). **(B)** CD8-positive cells in tumor mass (×200) (n=5 for each group). **(C)** The frequency CD3^+^CD8^+^ T cells in the spleen and lymph nodes (n=4 for each group). Data are expressed as the mean ± SEM. *p<0.05, **p<0.01 and ***p<0.001.

**Figure 3:**
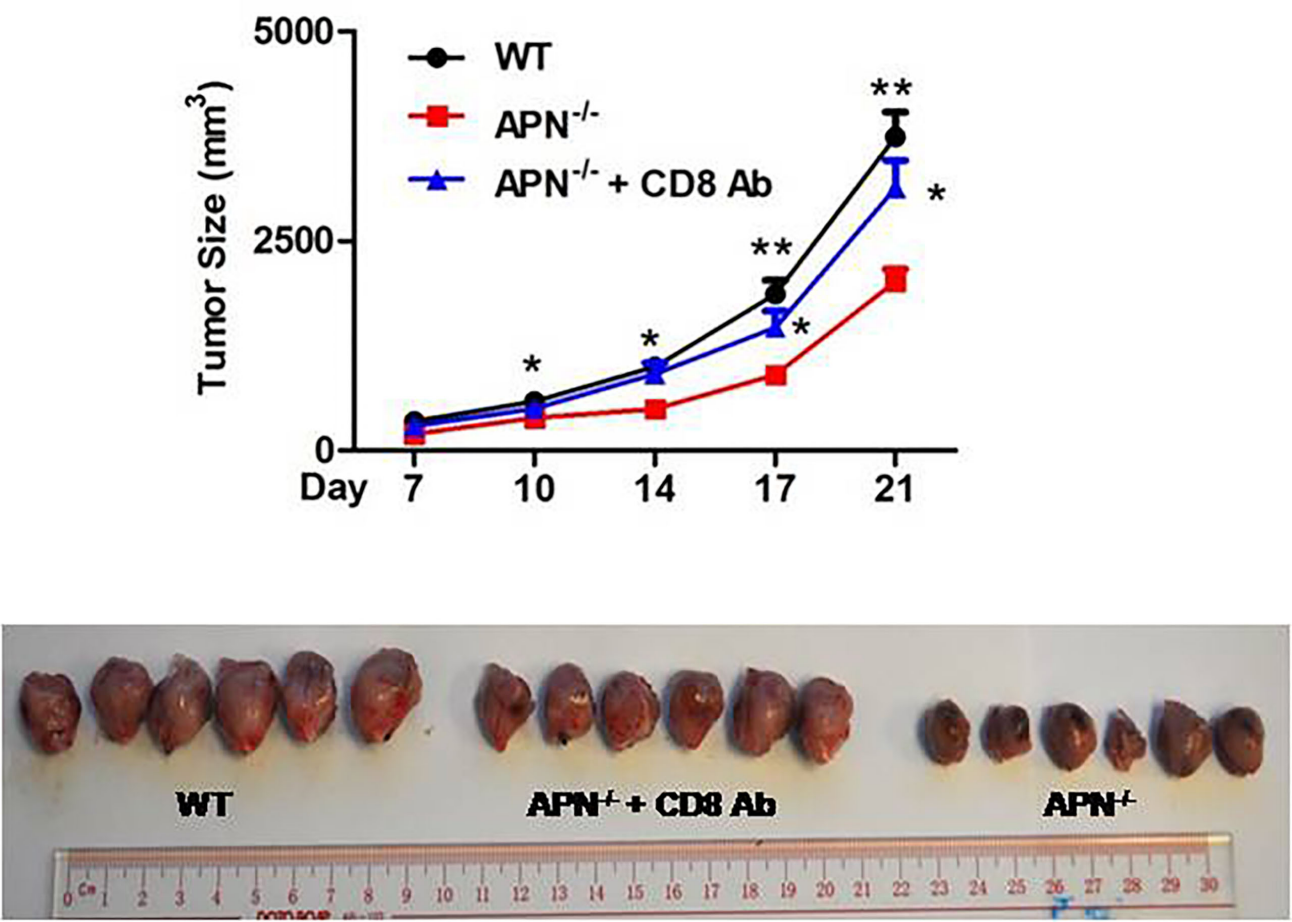
Blockage of CD8^+^ T cells impairs rhabdomyosarcom growth inhibition conferred by APN deficiency. MN/MCA1 cells were inoculated in APN^−/−^ mice, the CD8 mAb (5 μg/mice) were injected intraperitoneally to the mice every two days. Data are expressed as the mean ± SEM (n=6 for each group). Compared to APN^−/−^ mice, *p<.05 and **p<.01.

To investigate the role of APN in regulating the tumor-killing function of CD8^+^ T cells in tumors, a rhabdomyosarcoma model was established in both APN^−/−^ and wild-type mice by injecting the mice with MN/MCA1 cells. As expected, the tumor growth in APN^−/−^ group was significantly reduced (**Figure 2A**), and massive CD8^+^ T cell accumulation in the tumor mass, spleen, and lymph nodes was also observed in the APN^−/−^ group (**Figures 2B, C**). Subsequently, we blocked CD8^+^ T cells in tumor-bearing mice using CD8 mAb. As shown in **Figure 3**, the inhibitory effect of APN deletion on rhabdomyosarcoma growth was significantly abrogated after the blockade of CD8 mAb. These data suggest that APN deletion could negatively regulate the anti-tumor effect of CD8^+^ T cells to directly suppress rhabdomyosarcoma growth.

### APN Deficiency Enhances the Immune Response CD8^+^ T Cells

**Figure 4:**
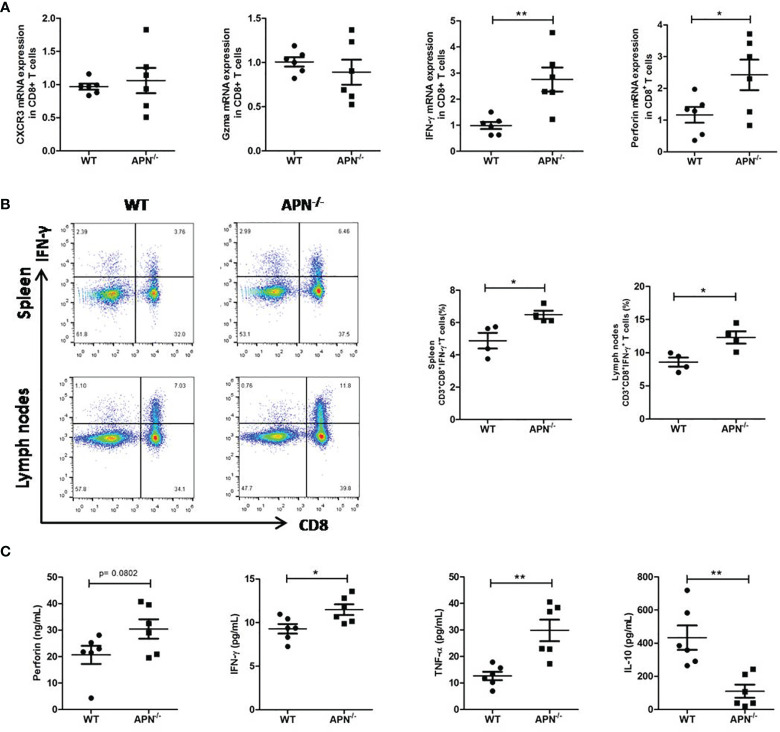
APN deficiency enhances CD8^+^ T cells immune response. **(A)** mRNA expression of IFN-γ, perforin, Gzma and CXCR3 in CD8^+^ T cells isolated from rhabdomyosarcom-bearing wild-type and APN^−/−^ mice (n=6 for each group). **(B)** The frequency CD3^+^CD8^+^IFN-γ^+^ T cells in the spleen and lymph nodes of rhabdomyosarcom-bearing wild-type and APN^−/−^ mice (n=4 for each group). **(C)** The levels of IFN-γ, perforin, TNF-α and IL-10 in the serum of rhabdomyosarcom-bearing wild-type and APN^−/−^ mice (n=6 for each group). Data are expressed as the mean ± SEM. *p<.05 and **p<.01.

We attempted to assess the immune status of CD8^+^ T cells in APN^−/−^ mice with rhabdomyosarcoma. The gene expression of IFN-γ, perforin, Gzma, and CXCR3 in CD8^+^ T cells isolated from both rhabdomyosarcoma-bearing wild-type and APN^−/−^ mice were quantified by RT-PCR. As shown in **Figure 4A**, CD8^+^ T cells isolated from rhabdomyosarcoma-bearing APN^−/−^ mice expressed relatively higher IFN-γ and perforin than those from rhabdomyosarcoma-bearing wild-type mice. Further, we detected CD8^+^IFN-γ^+^ T cells in the spleen and lymph nodes of both rhabdomyosarcoma-bearing wild-type and APN^−/−^ mice using flow cytometry. Consistent with the results of the RT-PCR, CD8^+^IFN-γ^+^T cells in the spleen and lymph nodes of rhabdomyosarcoma-bearing APN^−/−^ mice were markedly boosted, compared with CD8^+^IFN-γ^+^ T cells from rhabdomyosarcoma-bearing wild-type mice (**Figure 4B**). Subsequently, we determined the protein levels of IFN-γ, perforin, TNF-α, and IL-10 in the serum of both rhabdomyosarcoma-bearing wild-type and APN^−/−^ mice. As expected, compared to wild-type mice with rhabdomyosarcoma, the serum levels of IFN-γ, perforin and TNF-α of rhabdomyosarcoma-bearing APN^−/−^ mice were much higher, whereas the level of IL-10 in serum was much lower (**Figure 4C**). These findings indicate that APN deletion significantly enhances CD8^+^ T cell activation and cytotoxicity.

### APN Deficiency Inhibits STAT3 Activation in CD8^+^ T Cells

**Figure 5:**
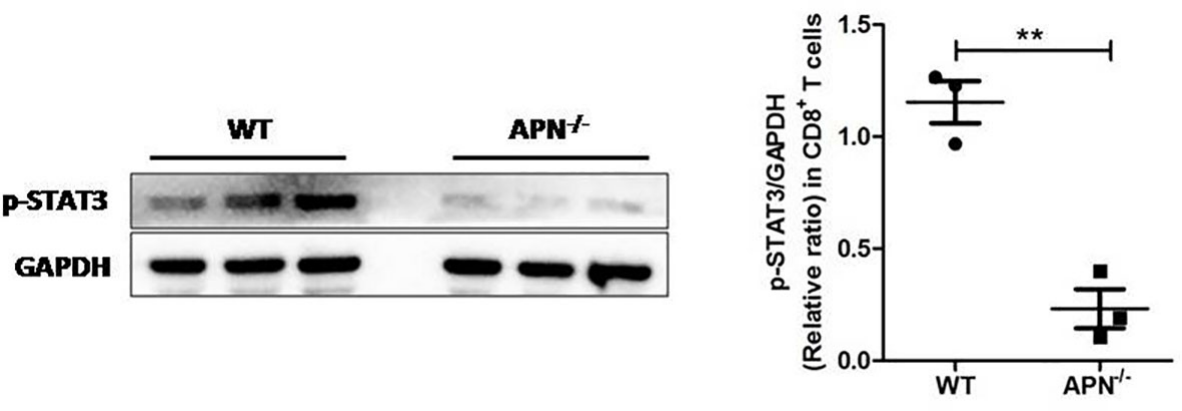
APN deficiency inhibits STAT3 activation in CD8^+^ T cells. MN/MCA1 cellswere inoculated in wild type (WT) and APN deficiency (APN^−/−^) mice, the CD8^+^ T cells were isolated from the spleen and lymph nodes after day 21. The expression of p-STAT3 was detected by western blot. Data are expressed as the mean ± SEM (n=3 independent mice for each group). **p<.01.

**Figure 6:**
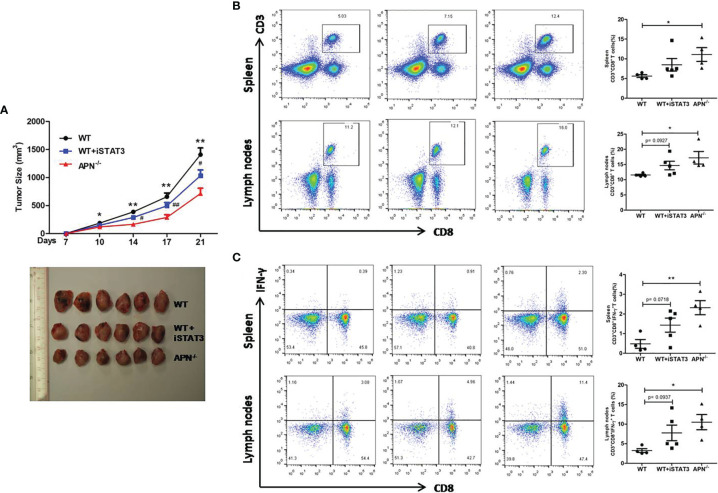
STAT3 inhibition suppresses rhabdomyosarcom growth and increases the accumulation of CD8^+^ and CD8^+^IFN-γ^+^ T cells in the spleen and lymph nodes. **(A)** Tumor size of wild type (WT), STAT3 inhibitor Stattic-treated WT (WT+iSTAT3) and APN deficiency (APN^−/−^) mice (n=6 for each group); compared to APN^−/−^ mice, *p < .05 and **p < .01; compared to WT mice, ^#^ *p* < .05 and ^##^ *p* < .01. **(B)** The frequency CD3^+^CD8^+^ T cells in the spleen and lymph nodes of WT, WT+iSTAT3 and APN^−/−^ mice (n=4-5 for each group). **(C)** The frequency CD3^+^CD8^+^IFN-γ+ T cells in the spleen and lymph nodes of WT, WT+iSTAT3 and APN^−/−^ mice (n=4-5 for each group). Data are expressed as the mean ± SEM. *p<.05 and **p<.01.

Since APN deletion greatly promotes CD8^+^ T cell activation and enhances its cytotoxicity, we then explored the underlying mechanism. As described previously, STAT3 activation has been shown to inhibit the antitumor functions of CD8^+^ T cells in various cancers (18, 19). We detected the protein expression of STAT3 in CD8^+^ T cells isolated from both rhabdomyosarcoma-bearing wild-type and APN^−/−^ mice. As shown in **Figure 5**, the phosphorylation of STAT3 in CD8^+^ T cells from APN^−/−^ mice with rhabdomyosarcoma was significantly suppressed compared to that in rhabdomyosarcoma-bearing wild-type mice, suggesting that APN deficiency inhibited STAT3 activation in CD8^+^ T cells. Subsequently, we administered the STAT3 inhibitor Stattic to rhabdomyosarcoma-bearing wild-type mice, and observed a remarkable tumor growth inhibition (**Figure 6A**), and the frequency of CD8^+^ as well as CD8^+^IFN-γ+ T cells in the spleen and lymph nodes were markedly upregulated (**Figures 6B, C**). These results all support the idea that APN deletion facilitates CD8^+^ T cell activation *via* inhibiting STAT3.

## Discussion

Tumors have specific mechanisms that disrupt the T-cell response. CD8^+^ T lymphocytes are central players in killing malignant cells in cancer immune responses. During tumor progression, CD8^+^ T lymphocytes are usually functionally dysfunctional and exhausted in the tumor immunosuppressive microenvironment. Therefore, exploring an effective way to recover the anti-tumor function of CD8^+^ T lymphocytes will contribute to the eradication of tumors. Our study clearly indicated that APN deficiency could enhance the immune response of CD8^+^ T lymphocytes to inhibit rhabdomyosarcoma growth by suppressing STAT3 activation.

APN was initially found to effectively regulate the immune response of innate immune cells in tumors. Tan et al. reported that APN retarded the maturation of dendritic cells and subsequent blunt antitumor immunity (7). Our previous study showed that APN deletion could reprogram tumor-associated macrophages into an M1-like phenotype to inhibit the growth of rhabdomyosarcoma (17). In this study, we found *in vitro* evidence supporting APN suppression of CD8^+^ T activation. APN deletion *in vivo* significantly promoted the accumulation of host CD8^+^ T cells in rhabdomyosarcoma tumor masses as well as in spleen and lymph nodes, and this antitumor effect could be abrogated by a CD8 mAb blockade. These findings indicate that APN deletion could negatively regulate the tumor-killing function of CD8^+^ T cells directly to suppress rhabdomyosarcoma. Notably, this is the first study to elucidate the regulatory effect of APN on CD8^+^ T lymphocytes.

It is well known that CD8^+^ T lymphocytes killing tumor cells are mediated by exocytosis using cytotoxic granules perforin and granzyme to directly destruct tumor cells, or by releasing cytokines, such as IFN-γ and TNF-α, to poison tumor cells indirectly (20). Our present study indicated that APN deficiency conferred a higher frequency of CD8^+^IFN-γ^+^ T cells in the spleen and lymph nodes, as well as higher levels of IFN-γ, perforin, and TNF-α and lower levels of IL-10 in the serum of rhabdomyosarcoma-bearing mice. These findings clearly indicate that APN deficiency greatly enhanced the capability of CD8^+^ T cells to kill tumor cells.

However, the underlying mechanism of APN deficiency in promoting CD8^+^ T cell activation and cytotoxicity remains to be explored. It has been reported that the expansion and cytolytic activity of CD8^+^ T cells are closely related to STAT3 activation. Kujawski et al. found that engineered STAT3^−/−^ CD8^+^ T cells were effective in infiltrating tumors and inhibiting their growth (21). Yue et al. further revealed that silencing STAT3 in T cells allows CXCR3 (the receptor for CXCL10) to be expressed in CD8^+^ T cells, leading to CD8^+^ T cells effectively accumulating at tumor sites (19). Moreover, Wang et al. reported that p-STAT3 was highly expressed on circulating CD8^+^ T cells in peripheral blood from hepatocellular carcinoma (HCC) patients. Highly expressed p-STAT3 was positively correlated to high levels of IL-4, IL-6, and IL-10, and negatively correlated to low levels of IFN-γ in the serum, which may contribute to immune surveillance disability against tumor cells (22). In the present study, we observed a lower expression of p-STAT3 in CD8^+^ T cells isolated from rhabdomyosarcoma-bearing APN^−/−^ mice, which was also accompanied by higher IFN-γ and lower IL-10 in the serum. We also found that inhibition of STAT3 activation *in vivo* by STAT3 inhibitor greatly increased CD8^+^ and CD8^+^IFN-γ^+^ T cells in the spleen and lymph nodes of rhabdomyosarcoma-bearing wild-type mice. These facts clearly indicate that the STAT3 signaling pathway may partly contribute to the effect of APN deficiency in promoting CD8^+^ T cell activation and cytotoxicity.

In summary, this study demonstrated that APN deficiency could promote CD8^+^ T cell activation and their cytotoxicity to kill tumors *via* inhibiting the STAT3 signaling pathway, and proved that targeting APN had potential in the treatment of rhabdomyosarcoma.

## Materials and Methods

### Animals and Tumor Models

C57BL/6 wild-type mice were purchased from Beijing Vital River Laboratory Animal Technology Co., Ltd. (Beijing, China). APN knockout mice with a C57/BL6 background were a kind gift from Prof. AM Xu (The University of Hong Kong). The mice were housed and bred in a specific pathogen-free/SPF environment with a 12-h inverted light–dark cycle at a temperature of 23 ± 2°C. Six-to-eight-week-old male offspring of C57BL/6 wild-type mice and APN^-/-^ mice were selected for the experiments. Soft tissue sarcoma was established as described in our previous study (17). In brief, 1×10^5^ MN/MCA1 cells were intramuscular injected into the caudal thigh muscle of mice. The tumor mass was measured, and the size was recorded twice a week. On day 21, the mice were sacrificed. The experimental protocol in this study was approved by the Animal Ethics Committees of Shenzhen University (Shenzhen, China) accordance to the National Institutes of Health guide for the Care and Use of Laboratory Animals (Registration No. 2018020).

### Purification and Stimulation of CD8^+^ T Cells

The CD8^+^ T cells were purified from the spleens of wild-type and APN^-/-^ mice after lysing red cells by positive selection using immunomagnetic beads according to the manufacturer’s instructions (CD8β [Ly-2] MicroBeads, Miltenyi). The purity of CD8^+^ T cells was verified above 95% cutoff by FACS determination using FITC-conjugated anti-CD8β antibody (eBioscience). Purified CD8^+^ T cells were stimulated with or without plate-bound anti-CD3 mAb (5 μg/mL) in combination with anti-CD28 mAb (1 μg/mL), in the presence or absence of APN (5μg/mL or a series of concentrations, as indicated).

### Immunomodulatory Antibody and Static Treatments

To examine the role of CD8^+^ T cells in APN deficiency-mediated inhibition of rhabdomyosarcoma growth, anti-mouse CD8α mAb (250 μg/mouse, BioXcell, West Lebanon, NH, USA) dissolved in 0.1 mL normal saline was intravenously injected into the tail of rhabdomyosarcoma-bearing APN^−/−^ mice every two days from day 7 to day 21. In the control mice, 0.1 mL of normal saline was injected. To determine the effect of blocking STAT3 signaling, STAT3 inhibitor stattic (10 mg/Kg, MCE, New Jersey, USA) dissolved in 0.25 mL normal saline containing 40% PEG400 and 1% dimethyl sulfoxide was intraperitoneally injected into rhabdomyosarcoma-bearing wild-type mice every two days from day 7 to day 21, according to previous reports (23, 24). In the control mice, 0.25 mL of normal saline containing 40% PEG400 and 1% dimethyl sulfoxide was injected.

### Cell Viability and Proliferation Assay

Purified CD8^+^ T cells from the spleen of wild-type mice were cultured in 96- or 24-well plates with a combination of plate-bound anti-CD3 mAb (5 μg/mL) and anti-CD28 mAb (1 μg/mL), in the presence of indicated concentrations of APN (0, 0.2, 1, and 5 μg/mL). The cells were then incubated for 24 h at 37°C and 5% CO_2_. Cell viability and proliferation were analyzed with a Cell Counting Kit-8 (CCK8, Beyotime, Shanghai, China) and a 5-bromo2-deoxy-uridine (BrdU) proliferation ELISA kit (Beyotime, Shanghai, China), respectively, according to the manufacturer’s protocols.

### Transwell Migration Assay

The migration assay was performed using a transwell chamber (8 µm; Corning). Purified CD8^+^ T cells (5×10^6^/well) from the spleen of wild-type mice were suspended in 100 µL medium were placed into the top chamber, and 600 µL medium containing APN (5 μg/mL) was added to the bottom well. After 24 h of incubation, cells in the bottom well were collected and counted using a Countess automated cell counter (Thermo Fisher Scientific).

### Immunohistochemistry Analysis

Immunohistochemistry was performed as previously described (17). In brief, paraffin sections of tumors were cut at 4 μm thickness and subjected to immunohistochemistry by heat-induced epitope retrieval with citrate buffer (0.01 M, pH 6.0). The samples were then treated with an endogenous peroxidase blocking solution (Dako Corp., Carpinteria, CA) and 10% goat serum to inhibit nonspecific binding. The tumor sections were incubated overnight with CD8 antibody at 4°C. The signals were detected using a VECTASTAIN Elite ABC Kit (Vector Laboratories, Burlingame, CA) according to the manufacturer’s instructions.

### Flow Cytometry Analysis

The spleens and lymph nodes were lightly ground between the frosted edges of two sterile glass slides, and the cell suspension was filtered through the 70-µm cell strainer. Immunosubsets from tumors, spleens, and lymph nodes were detected by flow cytometry (BD Biosciences, San Jose, CA). The frequency of CD8^+^IFN-γ^+^ T cells was assessed using intracellular cytokine staining. Purified cells were stimulated with PMA (10 ng/mL) and ionomycin (1 μg/mL) for 4 h and incubated for the last 1 h with brefeldin A (10 μg/mL). Cells were subjected to intracellular cytokine analysis using anti-IFN-γ antibody. The antibodies used for FACS included Pacific blue-conjugated anti-CD3, APC-conjugated anti-CD8, and PE-conjugated anti-IFN-γ (eBioscience). The data were analyzed using FlowJo software (TreeStar Inc., San Carlos, CA).

### RNA Extraction and RT-PCR Analysis

The total RNA of the CD8^+^ T cells was extracted using TRIzol^®^ (Invitrogen) RNA extraction protocol. cDNA was synthesized using a Reverse Transcription Kit (Takara, Osaka, Japan), and quantitative real-time PCR was performed on an ABI 7500 Real-Time PCR (RT-PCR) system using a SYBR Green Master Mix (Applied Biosystems, Foster, CA, USA). Relative quantification of mRNA expression was normalized with β-actin and analyzed using the delta-delta Ct (^2-ΔΔ^CT) method. Primer sequences (forward/reverse) were listed as follows: m-AdipoR1, forward 5′-ACG TTG GAG AGT CAT CCC GTA T-3′ and reverse 5′-CTC TGT GTG GAT GCG GAA GAT-3′; m-AdipoR2, forward 5′-GCC CAG CTT AGA GAC ACC TG-3′ and reverse 5′-GCC TTC CCA CAC CTT ACA AA-3′; m-IFN-γ, forward 5′- GCC ACG GCA CAG TCA TTG A-3′ and reverse 5′- TGC TGA TGG CCT GAT TGT CTT-3′; m-perforin, forward 5′- CAA GGT AGC CAA TTT TGC AGC-3′ and reverse 5′- GTA CAT GCG ACA CTC TAC TGT G-3′; m-CXCR3, forward 5′- TAC CTT GAG GTT AGT GAA CGT CA-3′ and reverse 5′- CGC TCT CGT TTT CCC CAT AAT C-3′; m-Gzma, forward 5′- TAC CTT GAG GTT AGT GAA CGT CA-3′ and reverse 5′-TGC TGCCCA CTG TAA CGT G-3′; m-β-actin, forward 5′-TGT CCA CCT TCC AGC AGA TGT-3′ and reverse 5′-AGC TCA GTA ACA GTC CGC CTA GA-3′.

### ELISA and Western Blotting

The levels of IFN-γ, perforin, TNF-α, and IL-10 in the serum were measured by ELISA (BD Bioscience, San Jose, CA) according to the manufacturer’s instructions. Western blotting was performed as previously described (23). Proteins were extracted from CD8^+^ T cells in the presence of a cocktail of phosphatase inhibitors and phenylmethylsulfonyl fluoride (PMSF) (Roche Applied Science, Branford, CT, USA), resolved,separated by 8–10% SDS-PAGE, and transferred to PVDF membranes. The membranes were then probed with the primary antibodies against p-STAT3 and GAPDH (Cell Signaling Technology, Beverly, MA, USA). Following incubation with the corresponding horseradish peroxidase-conjugated secondary antibodies, the protein bands were visualized with enhanced chemiluminescence reagents (Thermo Scientific, Rockford, IL, USA), detected using the ECL system, and the band intensity was measured using ImageJ software (National Institutes of Health, Bethesda, Maryland, USA).

### Statistical Analysis

GraphPad Prism 5.0 software (GraphPad Software Inc., San Diego, CA, USA) was used for all calculations. The results are expressed as the mean ± standard error of the mean (SEM). Comparisons between two groups were performed using a two-tailed Student’s t-test, and comparisons between multiple groups were performed usingone-way ANOVA, followed by Duncan’s multiple range tests. A p <.05 was considered statistically significant.

## Data Availability Statement

The original contributions presented in the study are included in the article/supplementary material. Further inquiries can be directed to the corresponding author.

## Ethics Statement

The animal study was reviewed and approved by Animal Ethics Committees of Shenzhen University.

## Author Contributions

HX: Conceptualization, writing-original draft, and funding acquisition. JP: Investigation and funding acquisition. HH: Investigation and analyzed data. QH: Investigation. CL, ZG, DH: Data analysis, methodology and resources. XS: Conceptualization and edited the manuscript. All authors contributed to the article and approved the submitted version.

## Conflict of Interest

The authors declare that the research was conducted in the absence of any commercial or financial relationships that could be construed as a potential conflict of interest.

## Publisher’s Note

All claims expressed in this article are solely those of the authors and do not necessarily represent those of their affiliated organizations, or those of the publisher, the editors and the reviewers. Any product that may be evaluated in this article, or claim that may be made by its manufacturer, is not guaranteed or endorsed by the publisher.
